# Currently available murine Leydig cell lines can be applied to study early steps of steroidogenesis but not testosterone synthesis

**DOI:** 10.1016/j.heliyon.2018.e00527

**Published:** 2018-02-01

**Authors:** Roger T. Engeli, Cornelia Fürstenberger, Denise V. Kratschmar, Alex Odermatt

**Affiliations:** Swiss Centre for Applied Human Toxicology and Division of Molecular and Systems Toxicology, University of Basel, Klingelbergstrasse 50, 4056 Basel, Switzerland

**Keywords:** Endocrinology, Pharmaceutical science

## Abstract

Androgen biosynthesis in males occurs to a large extent in testicular Leydig cells. This study focused on the evaluation of three murine Leydig cell lines as potential screening tool to test xenobiotics interfering with gonadal androgen synthesis. The final step of testosterone (T) production in Leydig cells is catalyzed by the enzyme 17β-hydroxysteroid dehydrogenase 3 (17β-hsd3). The endogenous 17β-hsd3 mRNA expression and Δ4-androstene-3,17-dione (AD) to T conversion were determined in the murine cell lines MA-10, BLTK1 and TM3. Additionally, effects of 8-Br-cAMP and forskolin stimulation on steroidogenesis and T production were analyzed. Steroids were quantified in supernatants of cells using liquid chromatography–tandem mass spectrometry. Unstimulated cells incubated with AD produced only very low T but substantial amounts of the inactive androsterone. Stimulated cells produced low amounts of T, moderate amounts of AD, but high amounts of progesterone. Gene expression analyses revealed barely detectable 17β-hsd3 levels, absence of 17β-hsd5 (Akr1c6), but substantial 17β-hsd1 expression in all three cell lines. Thus, MA-10, BLTK1 and TM3 cells are not suitable to study the expression and activity of the gonadal T synthesizing enzyme 17β-hsd3. The low T production reported in stimulated MA-10 cells are likely a result of the expression of 17β-hsd1. This study substantiates that the investigated Leydig cell lines MA-10, BLTK1, and TM3 are not suitable to study gonadal androgen biosynthesis due to altered steroidogenic pathways. Furthermore, this study emphasizes the necessity of mass spectrometry-based steroid quantification in experiments using steroidogenic cells such as Leydig cells.

## 1 Introduction

Steroid hormones are involved in the regulation of essential physiological processes. Systemic steroid levels are mainly produced by the adrenal glands synthesizing mineralocorticoids, glucocorticoids and precursors of active androgens, and the gonads synthesizing active sex steroids [1]. Disruption of steroidogenesis by genetic defects or environmental influences, including the exposure to synthetic chemicals, has been associated with developmental disturbances [2], impaired reproduction [3, 4], cancer [5, 6, 7], metabolic disorders [8, 9, 10], immune and neurologic diseases [11, 12].

For the identification of substances interfering with steroidogenesis and for mechanistic investigations, cell lines derived from the adrenals or from testicular Leydig cells represent alternatives to primary cells and animal experimentation that are ethically accepted, low-cost and rapid testing systems. Nevertheless, cell-based models must be well characterized and they should be used only for well-defined applications, including appropriate positive and negative controls. In contrast to the adrenals, where a human adrenal adenoma cell line (H295R) has been validated according to the Organization for Economic Cooperation and Development (OECD) guideline to detect substances disrupting steroid production [13], currently no human Leydig cell line to study testicular steroidogenesis is available. Human H295R cells are an appropriate model to test disruption of adrenal steroidogenesis. Unfortunately, this cell line is not suitable to study testosterone synthesis disruption due to low amount of total produced T and the lack of endogenous 17β-hydroxysteroid dehydrogenase type 3 (17β-HSD3) [14]. Moreover, the supplementation by Nu-serum for cultivation of H295R contributes considerable amounts of T and the low amounts produced by the cells are a result of the expression of AKR1C3 (17β-HSD5) rather than 17β-HSD3 [14]. Primary human and murine Leydig cells represent an ideal model to study disruption of T synthesis. However, primary cells are difficult to obtain due to low yield, and there are considerable inter-individual differences; similarly, the preparation of primary rodent Leydig cells is laborious and the yield rather low, thus limiting their use for medium to high throughput testing. There are, however, several commercially available rodent Leydig cell lines to study testicular steroidogenesis (reviewed in [15, 16]). Most of these cell lines have been developed decades ago and morphological and genetic transformations may have occurred in these lines over the years. MA-10 cells were derived from a spontaneous testicular murine tumor, which were subjected to *in vitro* immortalization [17]. TM-3 cells were derived from primary testicular murine cell cultures subjected to spontaneous immortalization *in vitro* [18]. Immortalized BLTK1 cells were established from a testicular tumor induced by the Simian virus T-antigen (SV40 TAG) oncogene of a transgenic mice expressing the inhibin-α promoter [19]. Forgacs et al. reported that BLTK1 cells display consistent induction of steroidogenesis for up to 30 passages [20].

Human and rodent *de novo* androgen production from cholesterol differs regarding Δ4-androstene-3,17-dione (AD) synthesis. In humans, AD is produced via the Δ^5^ metabolic steroid intermediates pregnenolone (Preg) and 17α-hydroxypregnenolone (17OH-Preg). The human enzyme CYP17A1 efficiently converts 17OH-Preg to dehydroepiandrosterone (DHEA) but has low affinity for 17α-hydroxyprogesterone (17OH-P). In rodents, CYP17 is able to convert Δ^4^ and Δ^5^ steroids, but in contrast to humans it prefers the Δ^4^ intermediates progesterone (P) and 17OH-P [21]. Importantly, in both human and rodents AD is converted in the last step to T by 17β-HSD3 [22].

Several reports describe the use of mouse Leydig cell lines to investigate the interference of xenobiotics with steroidogenesis, especially focusing on the disruption of T production (reviewed in [16]). Many studies have chosen a single steroid as a read-out, mostly T, and using antibody-based quantification methods. Such methods often suffer from limited specificity [23, 24, 25, 26], and it cannot be excluded that other steroid metabolites might interfere with the read-out due to the inability of antibodies to distinguish between structurally very similar steroid metabolites.

An initial aim of the present project was to identify a mouse Leydig cell model expressing substantial 17β-hsd3 levels in order to investigate the impact of substances on the last step of testicular T formation. Three mouse Leydig cell lines, MA-10, BLTK1 and TM3, were investigated by assessing the conversion of exogenous AD to T, the basal production of T, as well as the production of T and additional steroids following stimulation by 8-Br-cAMP and forskolin. The mRNA expression levels of key genes involved in androgen production was measured by quantitative RT-PCR, providing an explanation for the observed steroid production by these cells.

## 2 Materials and methods

### 2.1 Cultivation of MA-10, BLTK1 and TM3 cell lines

The mouse Leydig cell line MA-10 (ATCC, Manassas, VA, USA) was cultivated as described previously [27]. Cell culture materials and chemicals were obtained from Gibco, Carlsbad, CA, USA, and Sigma-Aldrich, St. Louis, MO, USA, unless otherwise stated. Briefly, cells were grown on 0.1% gelatin-coated cell culture dishes in DMEM/F12 medium containing 20 mM HEPES, pH 7.4, 15% horse serum, and 50 μg/mL gentamicin. MA-10 cells were exclusively used from passages 12 to 19. The BLTK1 mouse Leydig cell line (kindly provided by Prof. Ilpo Huhtaniemi and Dr. Nafis Rahman, University of Turku, Turku, Finland [20]) was maintained in DMEM/F12 medium with 10% fetal bovine serum (FBS), 100 U/mL penicillin and 100 μg/mL streptomycin. BLTK1 cells were exclusively used from passages 20 to 25. The mouse Leydig cell line TM3 was cultivated in DMEM/F12 medium, containing 15 mM HEPES, pH 7.4, 100 U/mL penicillin and 100 μg/mL streptomycin, 2.5 mM l-glutamine, 5% horse serum and 2.5% FBS. TM-3 cells were used from passages 11 up to 17. All cell lines were incubated under standard conditions (5% CO_2_, 37 °C). For ultra-pressure liquid chromatography–tandem mass spectrometry (UPLC–MS/MS) measurements phenol red-free medium containing overnight charcoal/dextran-treated FBS or horse serum was used.

### 2.2 Determination of mRNA expression

Total RNA from mouse Leydig cells (300,000 cells seeded in 6-well plates) was extracted using Trizol reagent, followed by reverse transcription using the Superscript III reverse transcriptase. The mRNA levels from different genes were analyzed using a Rotor-Gene 6000 light cycler (Corbett, Sydney, Australia). Reactions were performed in a total volume of 10 μL reaction buffer containing KAPA SYBR master mix (Kapasystems, Boston, MA, USA), 10 ng cDNA and specific oligonucleotide primers (Table 1). Relative gene expression was compared to the internal control cyclophilin A (Ppia).

**Table 1:** Oligonucleotide primers used for quantitative RT-PCR.

Gene	Oligonucleotide Forward Primers	Oligonucleotide Reverse Primers
***Hsd3b1***	5′-GTCACAGGTGTCATTCCCAG-3′	5′-TTCTTGTACGAGTTGGGCC-3′
***Cyp17a1***	5′-AGAGTTTGCCATCCCGAAG-3′	5′-AACTGGGTGTGGGTGTAATG-3′
***Hsd17b3***	5′-ACAATGGGCAGTGATTAC-3′	5′-GTGGTCCTCTCAATCTCTTC-3′
***Akr1C6***	5′-TCCGAAGCAAGATAGCAGATG-3′	5′-GTTGGACTATGTGGACCTGTAC-3′
***Srd5a1***	5′-TCACCTTTGTCTTGGCCTTC-3′	5′-TTATCACCATGCCCACTAACC-3′
***Akr1c14***	5′-GTACAAGCAAACACCAGCAC-3′	5′-ATGTCCTCTGAAGCCAACTG-3′
***Hsd17b1***	5′-GCTGTGTTGGATGTGAATGTG-3′	5′-ACTTCGTGGAATGGCAGTC-3′
***Ppia***	5′-CAAATGCTGGACCAAACACAAACG-3′	5′-GTTCATGCCTTCTTTCACCTTCC-3′

### 2.3 Determination of androstenedione metabolism

The conversion of radiolabeled AD by BLTK1 cells (10,000 grown in 24-well plates) was measured using a modified protocol from Legeza et al. [27]. Briefly, cells were incubated in serum-free DMEM/F12 medium containing 200 nM radiolabeled 50 nCi [1,2,6,7^−3^H]-AD (GE Healthcare, Little Chalfont, UK). The enzymatic reactions were terminated by adding 2 mM unlabeled AD and T dissolved in methanol. The steroids were separated on UV-sensitive silica TLC plates (Macherey-Nagel, Oensingen, Switzerland) using a chloroform-methanol solvent system at a ratio of 9:1. Bands migrating with an Rf of AD and T, as well as two fractions in between AD and T, were scraped off the TLC plate and transferred to tubes containing scintillation cocktail. Radioactive decay of AD and corresponding metabolites were analyzed using a scintillation counter (PerkinElmer, Waltham, MA, USA). For UPLC–MS/MS analysis cells were incubated with unlabeled AD and steroids formed were analyzed after extraction from bands of TLC plates by UPLC–MS/MS. Alternatively, for experiments where steroids were directly analyzed by UPLC–MS/MS, MA-10 (100,000), BLTK1 (200,000) and TM3 (75,000) cells were seeded in 12-well plates and cultivated for 24 h. Cells were washed with phosphate buffered saline (PBS) and incubated with charcoal/dextran-treated FBS/horse serum containing phenol red-free complete DMEM/F12 medium supplemented with AD, cAMP analogue or forskolin. Cells were incubated for 24 h, followed by collection of culture supernatants. Samples were stored at −20 °C prior to UPLC–MS/MS analysis.

### 2.4 Assessment of cross-reactivity of the enzyme immunoassay (EIA) kit for testosterone

The EIA kit for T (Cayman Chemical Company, MI, USA) was applied according to the manufacturer’s instructions. 5α-dihydrotestosterone (DHT) and 5β-dihydrotestosterone, androstenediol, 5α-androstanedione, androsterone (ADT), eticholanolone and DHEA were purchased from Steraloids (Newport, RI, USA). All tested metabolites were dissolved in ethanol, diluted in the buffer provided with the kit and measured at three different concentrations (150 pg/mL, 75 pg/mL and 37.5 pg/mL) in duplicates. Cross-reactivity was calculated in percentage of the recovery rate of the corresponding metabolite.

### 2.5 Ultra-pressure liquid chromatography–tandem mass spectrometry

Stock solutions for analyte standards (Steraloids) and their corresponding deuterium labeled internal standards (IS) (CDN-Isotopes, Pointe-Claire, Canada) were prepared in methanol at a concentration of 10 mM. Thereafter, standards and deuterium labeled IS working solutions were prepared by mixing each individual stock solution to obtain a working concentration of 100 μM. Calibration curves were prepared by serial dilution of the working solutions of standards in DMEM/F12 phenol red-free medium in the range of 0.975 nM–1000 nM. Cell supernatant was taken at the appropriate time point and IS at a final concentration of 0.1 μM in protein precipitation solution (zinc sulfate 0.8 M in water/methanol 50/50 v/v) were added. After shaking vigorously for 10 min at 4 °C, the samples were centrifuged for 10 min at 10,000 × *g*. Supernatants were transferred onto Oasis HBL SPE columns (Waters, Milford, MA, USA) that were preconditioned with methanol and water. Samples were washed twice with water, eluted with methanol and evaporated to dryness at a vacuum evaporator. Samples were reconstituted in 100 μL methanol by vigorously shaking for 30 min at 4 °C.

Analytes were measured simultaneously by UPLC–MS/MS using an Agilent 1290 UPLC instrument equipped with a binary solvent delivery system, an auto sampler (at 4 °C), and a column oven, coupled to an Agilent 6490 triple quadrupole mass spectrometer equipped with a jet stream electrospray ionization interface (AJS-ESI) (Agilent Technologies, California, USA). Fragmentation for multiple reaction monitoring (MRM) and source conditions within the positive ion mode were automated defined by use of the integrated compound- and source- optimizer software modules (Agilent Technologies, B.07.01). The general source parameters were set as following: Gas temperature 290 °C, gas flow 14 L/min, sheath gas temperature 350 °C, sheath gas flow 11 L/min, nozzle voltage 1500 V, cell accelerator voltage 5 V and fragmentation voltage 380 V. Specific optimized analyte conditions are shown in Table 2. Analyte separation was achieved using a reversed-phase column (ACQUITY UPLC BEH C18, 1.7 μm, 2.1 × 150 mm, Waters, Wexford, Ireland), heated to 65 ± 0.8 °C. The mobile phase consisted of water-acetonitrile-formic acid (A) (95/5/0.1; v/v/v) and (B) (5/95/0.1; v/v/v). The eluent gradient was set from 25 to 41.4% of B within 0–20 min using a ramping flow-rate from 0.65 mL/min to 0.36 mL/min, followed by a washout (100% of B, 20–22 min) and column re-equilibration (25% B1, 22–26 min) at a constant flow-rate of 0.65 mL/min. The injection volume was 5 μL per sample. Methanol in water (75/50 v/v) was used as needle and needle-seat flushing solvent for 10 s after sample aspiration. Samples were stored until analysis in the auto sampler (maintained at 4 °C). Data acquisition and analysis was performed using Mass Hunter Workstation Acquisition Software Version 07.01 SP1 and MassHunter Workstation Software Quantitative Analysis Version B.07.00/Build 7.0457.0, respectively (Agilent Technologies).

**Table 2:** Parameters for the androgens measured and mass spectrometer properties.

Steroid	RT [min]	Precursor Ion (m/z)	Product ion (m/z)	Collision energy (V)	Nebulizer (psi)	Ion Funnel RF high (V)	Ion Funnel RF low (V)	Dwell time (ms)	Capillary (positive, KV)
ADT	18.1	273.2	255.2	12	15	120	90	250	3
			147.1	24					
T	8.1	289.2	97.1	32	25	170	90	200	2.5
			109	28					
AD	9.5	287.2	97.1	20	15	170	90	100	2
			109	24					
DHT	12.5	291.2	159.1	24	15	120	90	100	3
			255	12					
ADT-d2	18.1	275.2	257.1	8	15	120	90	100	3
			118.1	52					
T-d2	8.1	291.5	111.1	13	25	170	90	100	2.5
			125	24					
AD-d7	9.4	294.2	113	28	15	170	90	100	2
			100	24					
DHT-d3	12.3	294.2	258.2	12	15	120	90	100	3
			162	24					

## 3 Results

### 3.1 Rapid metabolism of AD to androsterone by BLTK1 cells

**Fig. 1:**
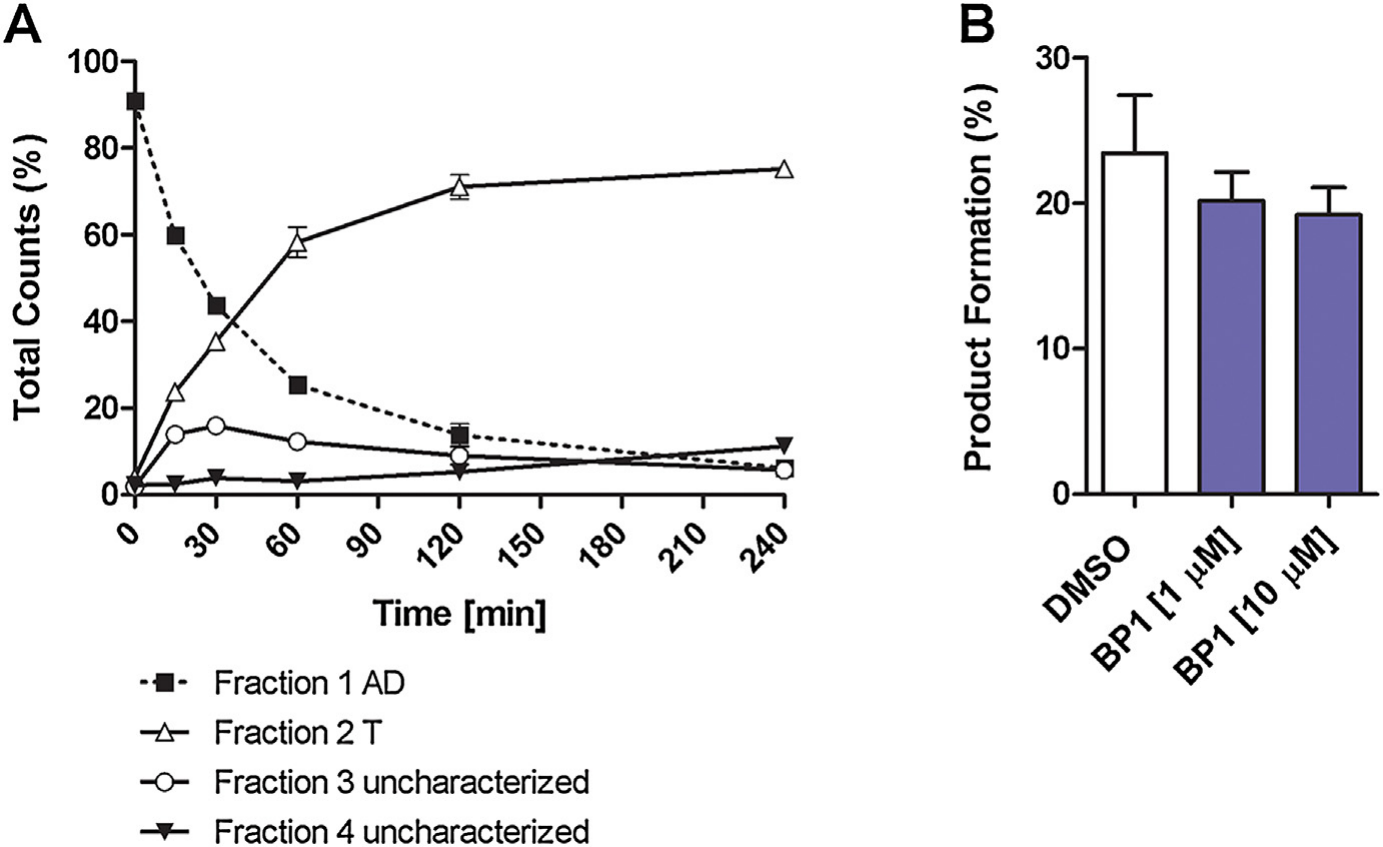
Metabolism of radiolabeled androstenedione in BLTK1 cells analyzed by TLC and scintillation counting. A, BLTK1 cells were incubated in serum-free medium supplemented with 200 nM AD containing 50 nCi [1,2,6,7-^3^H]-AD for up to 240 min, followed by separation of steroids by TLC and analysis of the excised fractions by scintillation counting. The main product formed was found in the fraction 2 migrating on TLC like T. Minor amounts of unknown products were detected in TLC fractions 3 and 4. Data represent mean ± SD from two independent experiments, each performed in duplicate, n = 4. B, Formation of the product migrating with an Rf like that for T by BLTK1 cells after 30 min exposure to 200 nM radiolabeled AD in the presence of vehicle control (0.1% DMSO) or the 17β-hsd3 inhibitor benzophenone 1 (BP1, 1 μM and 10 μM). Data represent mean ± SD from three independent measurements, each performed in duplicate, n = 6. In all experiments, the cell culture supernatants were collected and analyzed. Analytes were separated using TLC and samples were quantified using scintillation counting.

The initial aim of this study was to identify a mouse Leydig cell line suitable for assessing 17β-hsd3 expression and enzyme activity in order to investigate xenobiotics interfering with the last step of T synthesis. Based on a previous study reporting the expression of 17β-hsd3 and detecting T production using an antibody-based quantification method [20], the mouse Leydig cell line BLTK1 was first chosen as a cell model. Because mRNA levels often do not provide sufficient information on protein expression and a suitable, specific anti-17β-hsd3 antibody was not available, we attempted to measure 17β-hsd3 enzyme activity by incubating BLTK1 cells in serum-free medium supplemented with 200 nM of radiolabeled AD and determining its metabolism by subjecting the culture supernatants to steroid separation by TLC and analyzing the excised bands by scintillation counting. Four distinct fractions were collected: fraction 1 migrating with the Rf of AD, fraction 2 migrating with the Rf of T, and fractions 3 and 4 with Rf between AD and T. The results suggested a time-dependent metabolism of AD and the formation of one major metabolite migrating with the Rf of T, as well as two minor metabolites (Fig. 1A). T was expected to be the main product because of the rapid conversion of AD to T by 17β-hsd3 in adult Leydig cells. However, the formation of the main metabolite could not be blocked by the potent 17β-hsd3 inhibitor BP1 at concentrations of 1 μM and 10 μM (Fig. 1B), shown previously to efficiently inhibit 17β-hsd3-mediated T production in testis homogenates [28], indicating that another metabolite was formed. Therefore, the fraction migrating with the Rf of T was extracted from the TLC plate and subjected to UPLC–MS/MS analysis, revealing the absence of T.

**Fig. 2:**
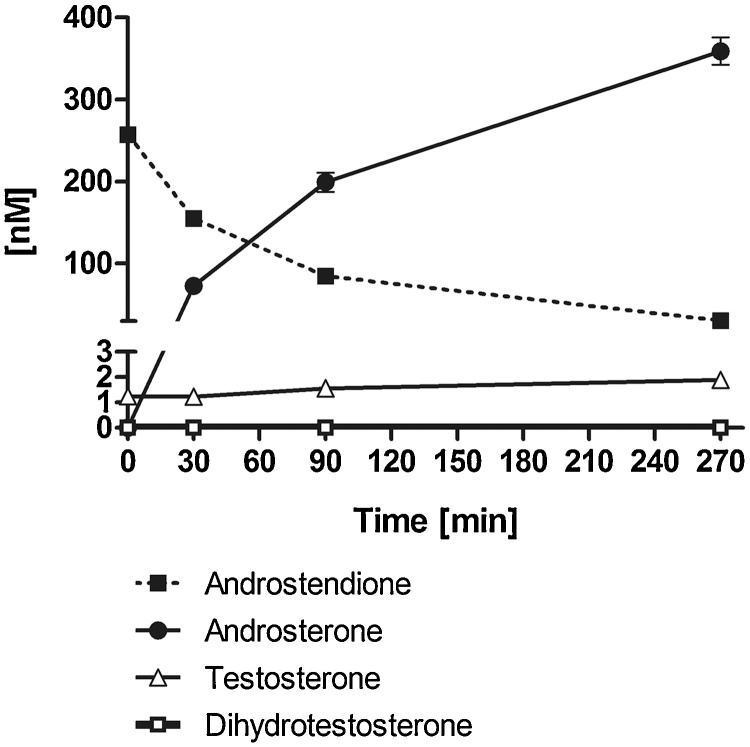
Time-dependent metabolism of androstenedione and formation of androsterone quantified by UPLC–MS/MS. BLTK1 cells were incubated in serum-free medium supplemented with 250 nM AD for different time (0, 30, 90, and 270 min), and the formation of ADT, T and DHT was quantified by UPLC–MS/MS in the cell culture supernatants. Data are shown as mean ± SD of technical triplicates.

Next, supernatants of BLTK1 cells incubated with 250 nM AD for different time periods were analyzed by UPLC–MS/MS. The main metabolite produced by BLTK1 cells was identified as ADT. The time-dependent loss of AD over time was proportional to the increase of ADT in the culture supernatants (Fig. 2). DHT was below the detection limit and only very low amounts of T could be measured.

### 3.2 Discrepancy to earlier work due to lack of specificity of antibody-based steroid quantification?

In an earlier study reporting T production by BLTK1 cells an EIA kit was applied for T quantification [20]. Radioimmuno assays (RIA) and enzymes-linked immunosorbent assays (ELISA) are methods that have been used to detect very low amounts of steroids for decades. However, all steroids share a common backbone and, therefore, it is important to assess the cross-reactivity rates between the key steroids produced by the system that is used in a corresponding experiment [24, 25, 29]. Thus, in the present study, the recovery rates of eight relevant androgenic steroids were tested by applying the T detection EIA kit (Cayman Chemical, Ann Arbor, MI, USA) that has been used in the earlier study reporting T production by BLTK1 cells [20]. The highest mean recovery rate was detected for DHT (59%) (Table 3). Other androgens showed recovery rates between 5–36%. Most importantly, cross-contamination was found for 5α-androstandione (21%), AD (25%) and ADT (16%). Thus, the high amounts of ADT formed by BLTK1 cells from AD, as shown in the present study, suggest that ADT might have been detected instead of T in the earlier study using this EIA kit for T quantification. These results emphasize the use of mass spectrometry-based steroid quantification methods when analyzing complex biological systems.

**Table 3:** Cross-reactivity of a commercially available testosterone ELISA kit (Cayman Chemical) toward several androgens. Experiments were performed according to the manufacturer’s protocol. Data show mean recovery rate of three tested steroid concentrations (150 pg/μL, 75 pg/μL, 37.5 pg/μL) from a representative experiment. Results were confirmed by a second independent experiment. Mean recovery rates for several tested androgens interacting with the anti-testosterone antibody varied from 5% up to 59%.

Steroid	Mean recovery rate (cross-reactivity)
Testosterone	100 ± 10
5α-Androstanedione	21 ± 8
5α-Dihydrotestosterone	59 ± 9
5β-Dihydrotestosterone	28 ± 5
Androstendione	25 ± 6
Androstenediol	36 ± 3
Androsterone	16 ± 11
Dihydroepiandrosterone	5 ± 3
Etiocholanolone	18 ± 1

### 3.3 Comparison of the conversion of androstenedione to testosterone and androsterone in MA-10 and BLTK1 cells

**Fig. 3:**
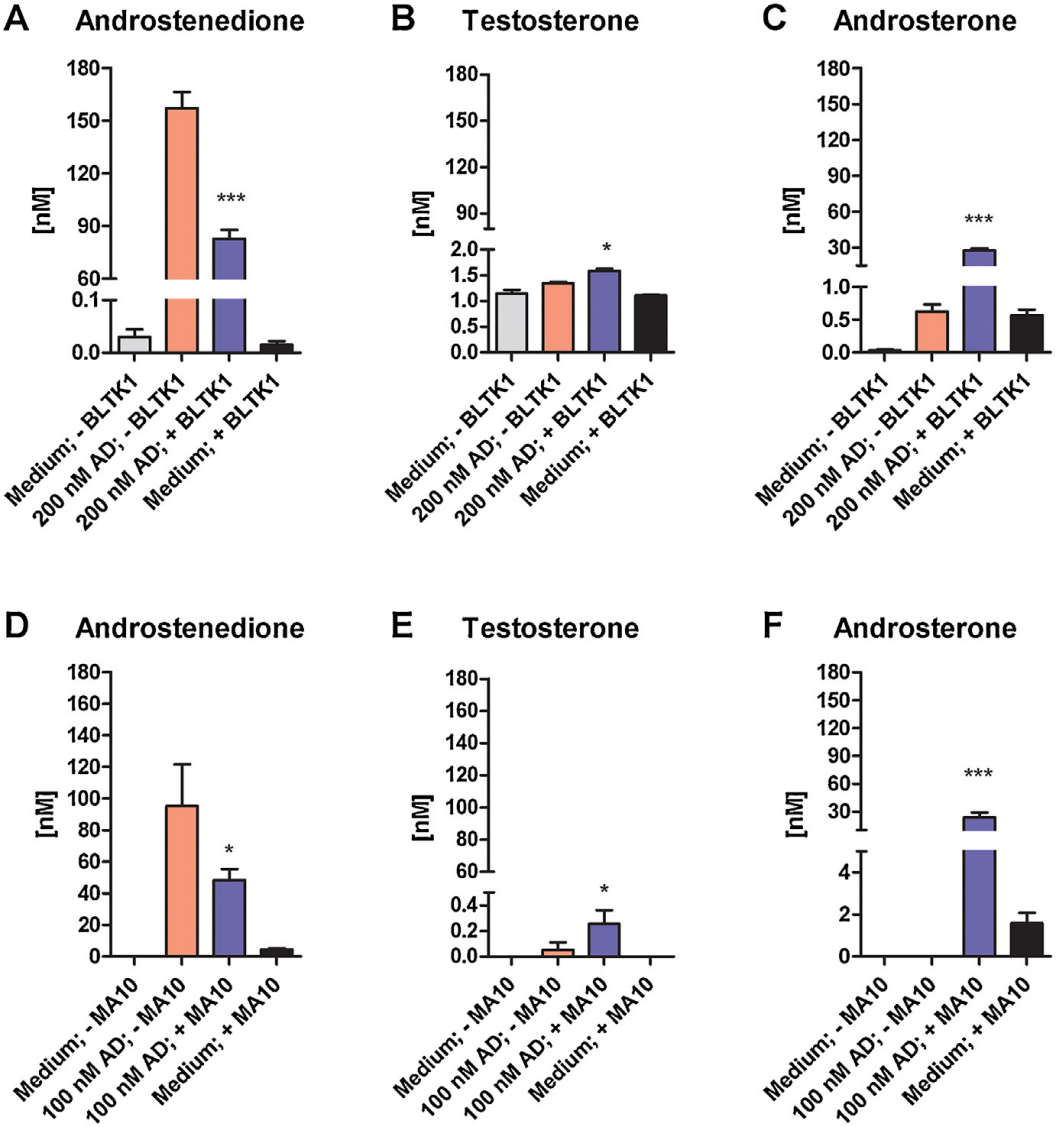
Formation of testosterone and androsterone in BLTK1 and MA-10 Leydig cells after incubation with androstenedione. Steroids were quantified by UPLC–MS/MS in supernatants of BLTK1 cells incubated with 200 nM AD (A–C) and of MA-10 cells incubated with 100 nM AD (D–F) in charcoal/dextran treated medium for 4.5 h. Results represent steroid concentrations (in nM) in supernatants of medium control (− cells), treatment control (200 or 100 nM AD, − cells), cells exposed to AD (200 or 100 nM AD, + cells), and cells incubated in medium in the absence of AD (medium only, + cells). Statistical significance was assessed using one-way ANOVA analysis followed by a post Tukey test. Data of BLTK1 cells represent mean ± S.D. from two independent experiments, each performed in duplicate, n = 4. MA-10 data represent mean ± S.D. from three independent experiments, each performed in duplicate, n = 6. *p < 0.05, ***p < 0.001.

**Fig. 4:**
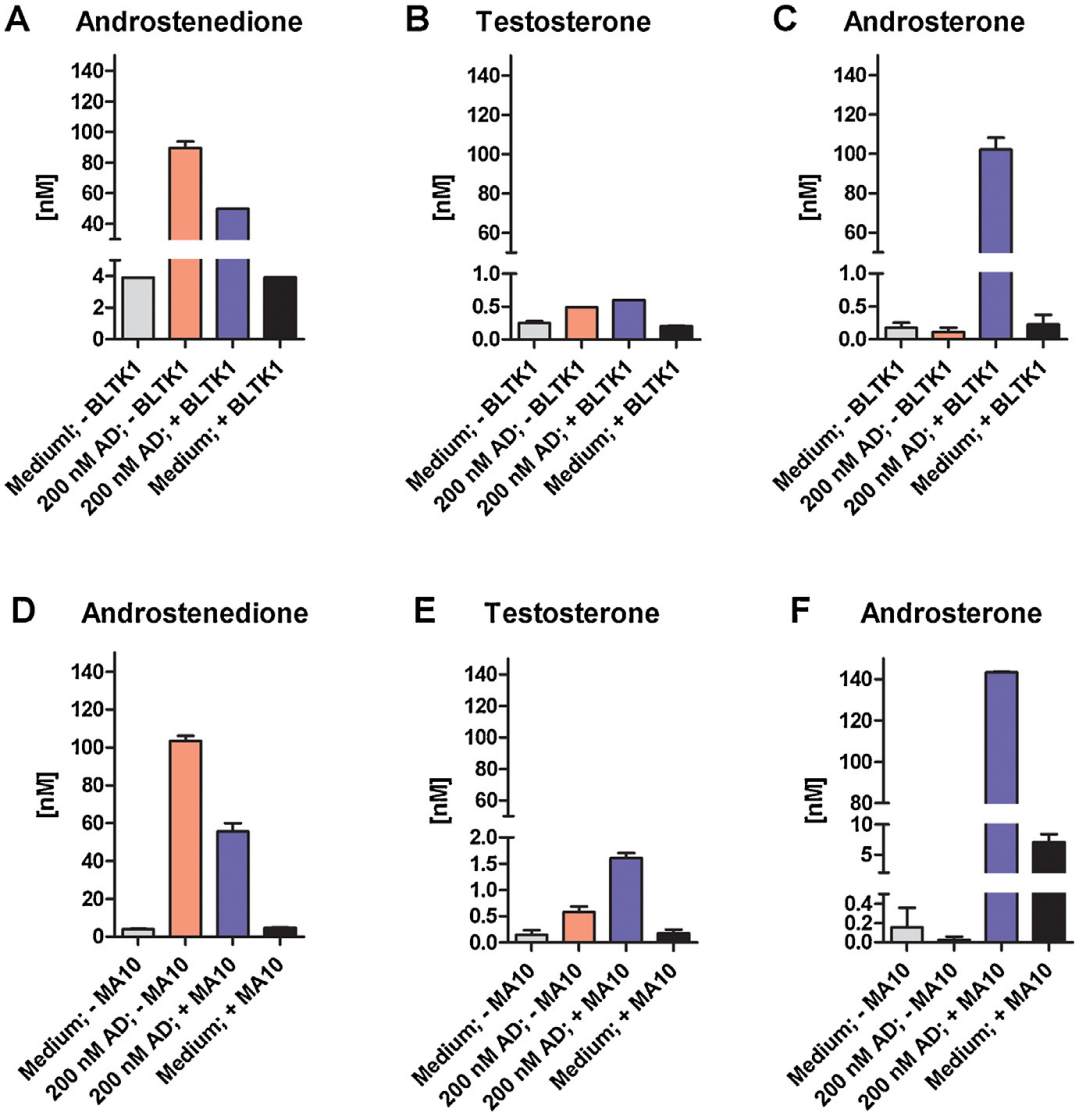
Formation of testosterone and androsterone in BLTK1 and MA-10 Leydig cells after incubation with androstenedione in serum-free medium. Before incubation with AD, BLTK1 and MA-10 cells were cultivated in serum-free media for 24 h. Steroids were quantified by UPLC–MS/MS in supernatants of BLTK1 cells incubated with 100 nM AD (A–C) and of MA-10 cells incubated with 100 nM AD (D–F) in serum-free medium for 4.5 h. Results represent steroid concentrations (in nM) in supernatants of medium control (− cells), treatment control (100 nM AD, − cells), cells exposed to AD (100 nM AD, + cells), and cells incubated in medium in the absence of AD (medium only, + cells). Data of BLTK1 and MA-10 cells represent mean ± S.D. from one experiment, each performed in duplicates.

Next, in an attempt to compare their capacity to catalyze the last step of T synthesis, BLTK1 and the more widely used MA-10 cells were incubated in charcoal/dextran treated medium supplemented with 200 and 100 nM AD, respectively, for 4.5 h (Fig. 3). AD concentrations dropped to about 50% after 4.5 h of incubation with both MA-10 and BLTK1 cells (medium control, +cells; Fig. 3A, D). BLTK1 cells did not produce AD *de novo* (Fig. 3A), compared to MA-10 cells, where low amounts of AD could be detected (Fig. 3D). AD was absent from the medium control in the absence of cells (Fig. 3A, D), indicating that it was completely removed by charcoal/dextran treatment. In contrast, T could not be completely removed from the BLTK1 culture medium, which contains charcoal/dextran treated FBS (Fig. 3B), whereas it was completely removed from horse serum containing MA-10 culture medium (Fig. 3E). Thus, higher amounts of T were measured in BLTK1 cell supernatants due to T contamination contributed by the serum. However, T concentrations in supernatants of both cell lines were increased after incubation with AD compared to the medium control (Fig. 3B, E), revealing a low conversion of AD to T in both cell lines. In contrast, ADT was a major metabolite formed upon incubation of both cell lines with AD (Fig. 3C, F). Both cell lines produced similar amounts of ADT from AD. Importantly, both BLTK1 and MA-10 cells incubated with AD produced approximately 50–100 times more ADT than T (Fig. 3C, F). Furthermore, MA-10 and BLTK1 cells both produced low amounts of ADT *de novo*. No ADT was detected in the medium controls in the absence of cells. However, regarding the mass balance, in both cell lines AD was not exclusively converted into ADT, indicating that either ADT is further metabolized (for example by conjugation) or that other metabolites were formed from AD. Interestingly, an additional experiment using BLTK1 and MA-10 cells incubated with serum-free medium showed a similar decrease in AD concentrations and the formation of very low amounts of T but high amounts of ADT (Fig. 4). Compared to cells incubated with charcoal/dextran treated serum, approximately 3–4 fold higher amounts of ADT were formed under serum-free conditions, emphasizing the importance of medium composition for steroidogenesis.

The absence of substantial production of T was further supported by the fact that 17β-hsd3 mRNA in BLTK1 and MA-10 cells is expressed at very low levels, 1000 times lower than in mouse testis (Table 4). This finding confirms that the endogenous expression of 17β-hsd3 in BLTK1 cells is too low to sufficiently convert AD into T. Therefore, the BLTK1 cell line is not a suitable model to study effects on T synthesis. Moreover, despite the high levels of Srd5a mRNA within both cell lines (data not shown) DHT is not produced, likely a result of the lack of functional 17β-hsd3.

**Table 4:** Comparison of mRNA expression levels of enzymes involved in androgen synthesis in mouse testis and Leydig cell lines. Testes were obtained from three different 11 weeks old C57bl6 mice. Testes were homogenized and lysed in TRI-reagent. Leydig cells were grown to confluence in 6-well plates and lysed using TRI-reagent. Messenger RNA was isolated and converted into cDNA. CT-values were determined using 10 ng of cDNA. Data on BLTK1 and TM3 were obtained from a single experiment performed in triplicate. Testis and MA-10 data were obtained from three independent experiments each performed in triplicate. Significant changes of mRNA expression in MA-10 cells compared to mouse testes are indicated. Testis *vs*. MA-10, *t*-test = ** *p* < 0.0001; * *p* < 0.01.

	Testis	MA-10	BLTK	TM3
*Hsd17b3*	22.4 ± 0.9	31.2 ± 0.2 **	31.36	n.d
*Akr1c14*	23.6 ± 0.7	17.3 ± 0.2 **	14.21	21.54
*Srd5a1*	21.8 ± 0.3	20.7 ± 0.5	19.77	25.05
*Hsd3b1*	17.6 ± 1.2	19.0 ± 0.5	12.35	23.31
*Cyp17a1*	17.4 ± 0.9	23.3 ± 1.0 *	25.98	28.55
*Hsd17b1*	26.9 ± 0.2	25.8 ± 0.2	25.32	26.78
*Hsd17b2*	22.2 ± 0.2	n.d	n.d	n.d
*Akr1c6*	n.d	n.d	n.d	n.d
*Ppia*	14.2 ± 0.1	13.6 ± 0.2	14.15	12.67

### 3.4 Effects on steroidogenesis in MA-10 cells by the inducers 8-Br-cAMP and forskolin

Forskolin and 8-Br-cAMP are well-known inducers of steroidogenesis *via* the cAMP-PKA pathway. Two different concentrations of each compound (10 and 50 μM) were used to stimulate steroid synthesis in MA-10 cells. Cells were treated with 10 and 50 μM of 8-Br-cAMP or forskolin for 24 h. Steroid concentrations in the cell supernatants were analyzed by UPLC–MS/MS.

**Fig. 5:**
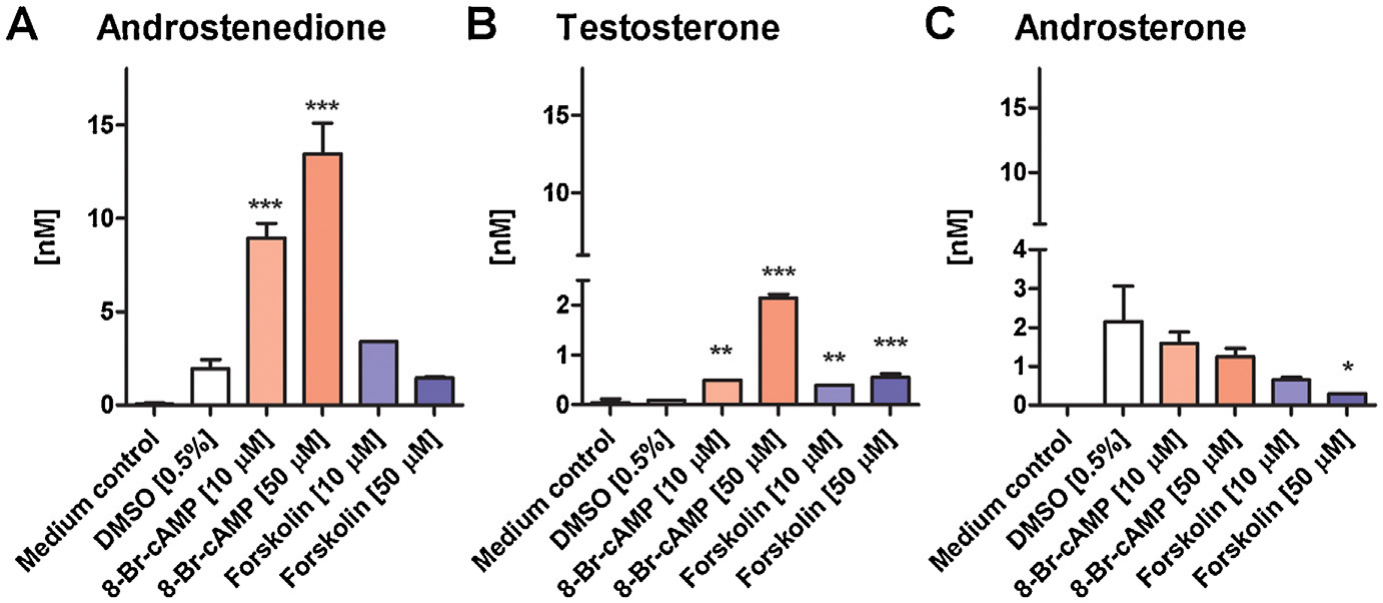
Quantification of *de novo* synthesis of androstenedione, testosterone and androsterone by MA-10 cells. MA-10 cells were incubated with 8-Br-cAMP or forskolin (10 μM and 50 μM) for 24 h, followed by quantification of steroids in supernatants by UPLC–MS/MS. DMSO (0.5%) and medium in the absence of cells served as controls. The medium contained charcoal/dextran-treated serum. Statistically significant difference to the DMSO control was assessed using one-way ANOVA analysis followed by a post Tukey test. Data represent mean ± S.D. from one out of four independent experiments performed in duplicate. *p < 0.05, **p < 0.01, ***p < 0.001.

As shown in Fig. 5A, 8-Br-cAMP concentration-dependently induced AD production in MA-10 cells. The results shown were obtained from a single experiment, which was independently reproduced four times. Comparable relative steroid values were observed in all four experiments; however, at different passages of the cells different absolute steroid levels were obtained. The inducible effect of 8-Br-cAMP was also found to be passage-dependent. Nevertheless, concentration-dependent stimulation of AD production was observed in every experiment. Treatment with forskolin did not result in significantly altered AD levels as measured in cell supernatants. Compared to the amount of AD detected in the cell supernatants, T levels were 10–20 times lower (Fig. 5B). In the basal state, the supernatants of the MA-10 cells vehicle control (DMSO 0.5%) contained less than 100 pM of T after 24 h of incubation. Despite the low concentrations detected, T was significantly increased by both inducers and at both concentrations. Interestingly, the 50 μM 8-Br-cAMP treatment was about four times more efficient than the 50 μM forskolin treatment in enhancing T production.

In contrast to T, significantly higher amounts of ADT were measured in MA-10 cells in the basal state (vehicle control, *i.e.* DMSO), reaching concentrations of about 2 nM (Fig. 5C). However, both treatments with 8-Br-cAMP and forskolin led to decreased levels of ADT in cell supernatants after 24 h, but only the values at 50 μM forskolin reached statistical significance, when compared to the DMSO control.

**Fig. 6:**
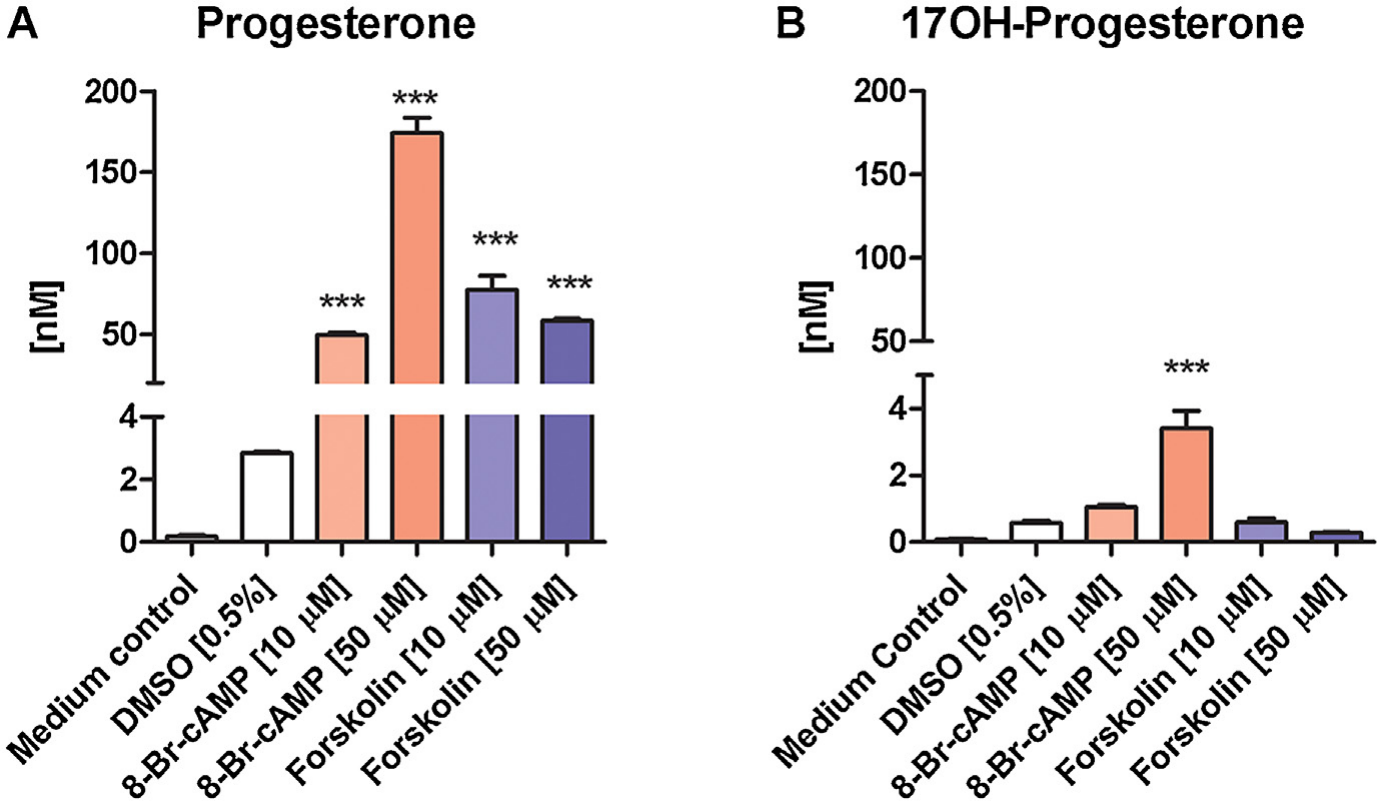
Quantification of *de novo* production of progesterone and 17-hydroxyprogesterone by MA-10 cells. Progesterone and 17-hydroxyprogesterone levels were quantified by UPLC–MS/MS in medium and supernatants of MA-10 cells stimulated for 24 h with 8-Br-cAMP or forskolin (10 μM and 50 μM). The medium contained charcoal/dextran treated serum. DMSO (0.5%) and medium in the absence of cells served as controls. Statistical differences to the DMSO control were assessed using one-way ANOVA analysis followed by a post Tukey test. Data represent mean ± S.D. from one out of three independent experiments performed in duplicate. *** *p* < 0.001.

Cells in the basal state (vehicle control) produced P to reach a concentration of 3 nM after 24 h. P levels were significantly increased in supernatants of cells stimulated with 8-Br-cAMP or forskolin (Fig. 6A). 17OH-P levels were increased by 20–50 fold in supernatants of cells stimulated with 50 μM 8-Br-cAMP but not at the lower concentration of 10 μM or in the presence of forskolin (Fig. 6B).

### 3.5 Analysis of the expression of genes involved in androgen synthesis

In order to understand the reason for the inability of the mouse MA-10 Leydig cell line to produce substantial amounts of T, the mRNA expression of several genes with a key role in androgen synthesis was determined and compared with the corresponding levels in mouse testis. For comparison, BLTK1 and TM3 were also qualitatively assessed in a single experiment. Ppia served as a house-keeping control. Importantly, 17β-hsd3, essentially catalyzing the last step of T synthesis, was well expressed in mouse testis but not detectable in TM3 and only at very low levels in MA-10 and BLTK1 cells (Table 4). Akr1c6, also known as 17β-hsd5 (and corresponding to AKR1C3 in human), was not expressed at detectable levels in mouse testis and in any of the three cell lines. Mouse 17β-hsd1, which can accept AD as a substrate to form T [30], was found to be expressed in mouse testis as well as in all three mouse Leydig cell lines, providing an explanation for the low levels of T formed by these cell lines despite of the absence or background levels of 17β-hsd3 and the absence of Akr1c6. Interestingly, 17β-hsd2, which converts estradiol to estrone but also was reported to be able to convert 5α-DHT to 5α-androstanedione and T to AD [31, 32], was expressed in mouse testis but not in any of the three cell lines.

An efficient androgen production depends on the expression of 3β-hsd1 and Cyp17a1, which were both well expressed in mouse testis. The lower expression of Cyp17a1 in the Leydig cell lines compared to mouse testis explains the preferred production of P in the three cell lines. The considerable amounts of ADT generated by MA-10 and BLTK1 cells are likely a result of the high expression of Srd5a1 (5α-reductase) and Akr1c14 (3α-HSD).

## 4 Discussion

This study investigated the capability of three commonly used mouse Leydig cell lines, *i.e.* TM3, BLTK1 and the most widely used MA-10, to synthesize T in their basal state or upon stimulation by 8-Br-cAMP or forskolin. All three cell lines produced no or very low amounts of T in their basal state. Nevertheless, in MA-10 cells T synthesis was detectable but it remained an order of magnitude lower than the production of AD and two orders of magnitude lower than that of P upon stimulation by 8-Br-cAMP or forskolin. This data is in line with previous reports by Lacroix et al., reporting negligible T production due to decreased 17β-hydroxysteroid dehydrogenase activity in the P variant of the Leydig cell tumor (M5480), adapted for serial transplantation by Dr. W. F. Dunning (Miami) to establish the MA-10 cell line. Real-time PCR analyses in this study revealed very low or absent 17β-hsd3 mRNA expression levels in MA-10, BLTK1 and TM-3 cells. The enzyme 17β-hsd3 essentially catalyzes the final step of T synthesis in Leydig cells and, therefore, these cell lines are not suitable as screening tool for T synthesis disruption. A variant of the M5480 Leydig cell tumor (M5480A) producing similar amounts of P and T at basal levels was reported [33]. This variant might be used to develop a murine Leydig cell line for investigations into the disruption of T generation and the regulation of the 17β-hsd3 expression and enzyme activity.

Other investigators reported increasing amounts of T in supernatants of BLTK1 cells that were stimulated with recombinant human chorionic gonatropin (rhCG) [20, 34, 35]. However, they also observed that BLTK1 cells produced much more P than T, consistent with the present study. Nevertheless, these investigators did not mention whether they used serum-free media or charcoal treated media in their experiments, and no data on cross-reactivity assessment of the ELISA kits used were reported; thus an over estimation of the reported T generation in these earlier studies cannot be excluded.

**Fig. 7:**
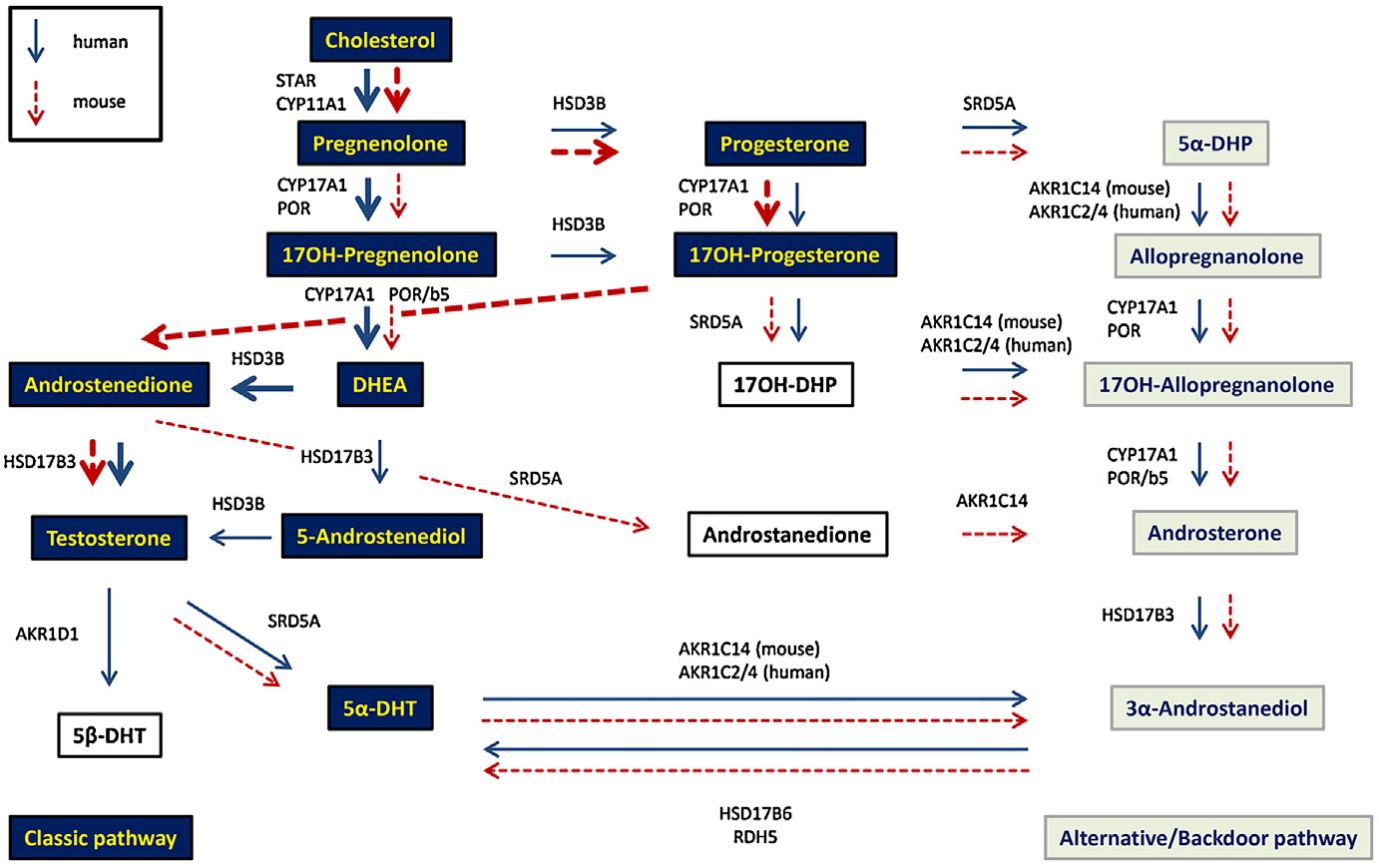
Schematic representation of murine and human gonadal androgen synthesis. Classic murine and human biosynthesis pathways of T in testis (black background). Alternative/backdoor pathway of 3α-androstanediol in human and mouse (grey background). Intermediate steroids are shown in boxes on white background. Murine pathways are depicted by dotted and human pathways by black arrows. The main human biosynthesis pathway of T via DHEA is shown by a bold black arrow. Preferred murine pathways of T via P and 17OH-P are shown by bold dotted arrows [36, 56].

Unexpectedly, the addition of exogenous AD to BLTK1 and MA-10 cells resulted in only very low amounts of T formation but considerable generation of ADT. An explanation for this observation was provided by the mRNA expression analysis revealing high expression of Akr1c14 (3α-hsd) and Srd5a1 (5α-reductase), two key enzymes required for the conversion of AD to ADT via the intermediate 5α-androstanedione (Fig. 7) [36, 37, 38]. Additionally, the known 17β-hsd3 inhibitor BP-1 was not able to inhibit the low amounts of T generated by MA-10 cells, and the 17β-hsd3 mRNA expression was very low, while 17β-hsd1 was well expressed, suggesting that T synthesis unlike in testis, was not mediated by 17β-hsd3 but rather by 17β-hsd1. It has been shown earlier that murine 17β-hsd1 can convert AD to T [30]. This is important regarding the interpretation of results obtained from investigations into effects of xenobiotics and endogenous mediators on T synthesis by these murine Leydig cell lines.

An important issue remains the analytical method chosen for T quantification. Since antibody-based quantification methods often are limited regarding their specificity [24], such methods should mainly be applied for applications where the identity of the generated steroids is known, and mass spectrometry-based methods should be used for the analysis of samples with complex steroid patterns. As shown in this study, the explicit use of the EIA kit for T quantification would have led to the misinterpretation that mainly T instead of ADT is formed from AD by MA-10 and BLTK1 cells. Also, the use of radiolabeled steroids, followed by separation by TLC, could result in misleading conclusions if several metabolites are formed and identical standards are not included as controls.

Several earlier studies applied MA-10 [39, 40, 41, 42, 43, 44, 45, 46, 47], BLTK1 [20, 35], or TM3 cells [48, 49, 50, 51] to analyze effects of endogenous and exogenous substances on steroidogenesis by measuring T production using antibody-based methods. In most of these studies there is only limited information available on the specificity of the antibody used, and the commercial suppliers mostly included only a limited number of steroid metabolites in their characterization. Thus, it cannot be excluded that the reported concentrations were overestimated due to cross-reactivity. In many of the above studies, the steroid concentrations in the medium used for the experiment are not explicitly defined, and different medium/serum combinations were used in different studies, making a direct comparison difficult.

The present study indicates that BLTK1 and MA-10 cells in the presence of medium containing charcoal/dextran treated serum mainly metabolize exogenously added AD to ADT, and that ADT formation is even more pronounced under serum-free conditions. The results also emphasize that care should be taken regarding the conclusions drawn on altered T production in experiments using these murine Leydig cell lines because 17β-hsd3 seems not to be functionally expressed and T formation probably is a result of 17β-hsd1 expression in these cells. Thus, a suitable Leydig cell line expressing all key gonadal steroidogenic enzymes is still needed. The human adrenal H295R cell line is a well-established model to test endocrine disrupting chemicals. However, this cell line is also not suitable to study T synthesis disruption due to low amounts of totally produced T and the lack of endogenous 17β-HSD3. Thus, any AD into T conversion observed in these cells most likely is a result of the ubiquitously expressed AKR1C3 (17β-HSD5) [14]. Thus, human or mouse primary Leydig cells are currently the only reliable model to investigate 17β-hsd3 activity and its regulation as well as disruption of T synthesis.

Gene expression analysis further revealed that 3β-hsd1 was well expressed but Cyp17 was substantially lower in the murine Leydig cell lines compared to adult mouse testis, providing an explanation for the production of low amounts of androgens but high P. The fact that Srd5a1 and Akr1c14 are highly expressed in these cell lines explains the favored synthesis of ADT and other steroid metabolites of the backdoor pathway (Fig. 7).

Unfortunately, the three murine Leydig cell lines BLTK1, MA-10, and TM3 show a fetal Leydig cell-like phenotype lacking 17β-hsd3 expression. Shima et al. showed that fetal Leydig cells produce and secrete AD, while T is further produced by Sertoli cells. The fetal testis produces much less T than adult Leydig cells [52, 53]. O’shaughnessy et al. showed that 17β-hsd3 was expressed in seminiferous tubules (Sertoli cells) up to day 20, and later on 17β-hsd3 expression was restricted to the interstitial tissue in Leydig cells [54]. Mahendroo et al. showed that the predominant androgen in immature mouse testis is 3α-androstanediol (3α-diol) [55]. In our experiments, forskolin significantly decreased ADT concentrations in supernatants of MA-10 cells. This may be due to forskolin-mediated stimulation of the production of steroids involved in the backdoor pathway, leading to the accumulation of 3α-diol (Fig. 6). A prominent peak was detected in supernatants of forskolin treated cells using LC–MS/MS that is expected to be 3α-diol; however, verification using an authentic standard will be required.

This study highlights the limitations of three murine Leydig cells as potential screening tool to investigate the endogenous disruption of T synthesis. Nevertheless, these cell lines are still suitable to examine testicular disruption of the steroidogenic pathway up to P production, which seems to be intact and can be stimulated using 8-Br-cAMP and hcG. Importantly, steroid levels should be measured using a validated and accurate quantification such as LC–MS/MS or GC–MS/MS in order to minimize over- or underestimation of the quantified steroid concentrations.

## 5 Conclusion

The three murine Leydig cell lines MA-10, BLTK1 and TM3 produce no or only very low T under basal conditions. T production is measurable in MA-10 cells and is stimulated by 8-Br-cAMP; however, under these conditions one and two orders of magnitude more AD and P, respectively, are produced. The T formed by these cell lines is probably due to 17β-hsd1 expression because 17β-hsd3 expression is barely detectable and inhibition of this enzyme did not abolish T formation. Furthermore, exogenous AD was predominantly metabolized to ADT and only minor amounts of T were formed. Thus, MA-10, BLTK1 and TM3 cell lines are not suitable models to investigate interferences by substances of Leydig cell T synthesis. Mouse and human Leydig cell lines expressing the entire set of steroidogenic enzymes should be developed for high throughput screening of potential endocrine disrupting chemicals. Moreover, this study further emphasizes the need for replacement of antibody-based steroid quantification methods by mass-spectrometry-based methods (GC–MS/MS, LC–MS/MS) when analyzing samples from biological systems producing several steroid metabolites.

## Declarations

### Author contribution statement

Roger T. Engeli: Conceived and designed the experiments; Performed the experiments; Analyzed and interpreted the data; Wrote the paper.

Cornelia Fürstenberger: Conceived and designed the experiments; Performed the experiments; Analyzed and interpreted the data.

Denise V. Kratschmar: Performed the experiments; Analyzed and interpreted the data.

Alex Odermatt: Conceived and designed the experiments; Analyzed and interpreted the data. Contributed reagents, materials, analysis tools or data; Wrote the paper.

### Competing interest statement

The authors declare no conflict of interest.

### Funding statement

This work was supported by the Swiss National Science Foundation (31003A-159454), the Novartis Research Foundation, and the Swiss Centre for Applied Human Toxicology.

### Additional information

No additional information is available for this paper.
